# Supplementary material for: Comparative Chloroplast Genome Study of Zingiber in China Sheds Light on Plastome Characterization and Phylogenetic Relationships
Source: Genes (Basel). 2024 Nov 19;15(11):1484. doi: 10.3390/genes15111484 (PMC11593964; doi:10.3390/genes15111484)
Supplement: Supplementary file 1 [file genes-15-01484-s001.zip › genes-3312538-supplementary.pdf]

Table S1. Information of plastome sequences downloaded from NCBI.

Family	Tribe	Taxon	GenBank accession	Genome size
**Cannaceae**		*Canna indica*	KF601570	162,182
**Costaceae**		*Costus pulverulentus*	KF601573	163,239
		Hellenia viridis	MK262733	168,966
**Musaceae**		*Ensete glaucum*	LC610748	168,811
		Musa itinerans	NC_035723	168,985
		Musella lasiocarpa	NC_035637	169,178
**Zingiberaceae**	**Alpinieae**	*Alpinia chinensis*	NC_050165	163,590
		Alpinia hainanensis	MK262728	162,387
		Amomum krervanh	MF991963	162,766
		Lanxangia tsaoko	MK926774	163,648
		Wurfbainia villosa	MH161416	164,068
	**Zingibereae**	*Boesenbergia kingii*	MW326451	163,198
		Cautleya gracilis	NC_046845	164,001
		Curcuma involucrata	MK262725	163,300
		Curcuma longa	MK919702	159,550
		Hedychium coronarium	MK262736	163,949
		Hedychium spicatum	NC_047248	163,531
		Kaempferia elegans	MK209002	163,555
		Kaempferia galanga	MK209001	163,811
		Monolophus coenobialis	MW326458	162,136
		Roscoea alpina	MT012418	163,395
		Roscoea humeana	NC_046582	160,288
		Zingiber corallinum	MW801385	160,957
		Zingiber densissimum	OP869975	163,607
		Zingiber ellipticum 5	OP869976	163,455
		Zingiber flavomaculosum	OP869987	163,298
		Zingiber koshunense	OP869977	163,394
		Zingiber leptorrhizum	OP869984	162,956
		Zingiber mioga 2	MW067011	159,868
		Zingiber montanum 1	OP869985	163,476
		Zingiber montanum 2	MK262727	164,464
		Zingiber montanum 3	MW801386	161,483
		Zingiber neotruncatum	OP869978	162,481
		Zingiber officinale 4	MW602894	162,721
		Zingiber officinale 5	NC_044775	162,621
		Zingiber orbiculatum	OP869979	163,527
		Zingiber purpureum 2	OP869980	163,135
		Zingiber recurvatum 3	MT473712	163,151
		Zingiber smilesianum	OP869981	163,640
		Zingiber spectabile	JX088661	155,890
		Zingiber striolatum 2	ON646165	163,711

	Zingiber teres	OM868074	163,428
	Zingiber xishuangbannaense	OP869982	163,487
	Zingiber yingjiangense	OP869983	163,623
	Zingiber zerumbet	MK262726	163,589

Table S2. Gene composition of *Zingiber* species. * Genes including a single intron. ** Genes including two introns. (×2) Genes duplicated in the inverted repeat regions.

Category for genes	Group of genes	Name of genes
Photosynthesis	Subunits of photosystem I	*psaA*, *psaB*, *psaC*, *psaI*, *psaJ*
	Subunits of photosystem II	*psbA*, *psbB*, *psbC*, *psbD*, *psbE*, *psbF*, *psbH*, *psbI*, *psbJ*, *psbK*, *psbL*, *psbM*, *psbN*, *psbT*, *psbZ*
	Subunits of cytochrome b/f complex	*petA*, *petB**, *petD**, *petG*, *petL*, *petN*
	Subunits of ATP synthase	*atpA*, *atpB*, *atpE*, *atpF**, *atpH*, *atpI*
	Subunits of NADH dehydrogenase	*ndhA**, *ndhB**(×2), *ndhC*, *ndhD*, *ndhE*, *ndhF*, *ndhG*, *ndhH*, *ndhI*, *ndhJ*, *ndhK*
	Subunit of rubisco	*rbcL*
Self-replication	RNA polymerase	*rpoA*, *rpoB*, *rpoC1**, *rpoC2*
	Large subunit of ribosomal proteins	*rpl2**(×2), *rpl14*, *rpl16a*, *rpl20*, *rpl22*, *rpl23*(×2), *rpl32*, *rpl33*, *rpl36*
	Small subunit of ribosomal proteins	*rps2*, *rps3*, *rps4*, *rps7*(×2), *rps8*, *rps11*, *rps12***(×2), *rps14*, *rps15*, *rps16**, *rps18*, *rps19*(×2)
	Ribosomal RNAs	*rrn4.5*(×2), *rrn5*(×2), *rrn16*(×2), *rrn23*(×2)
	Transfer RNAs	*trnA-UGC**(×2), *trnC-GCA*, *trnD-GUC*, *trnE-UUC*, *trnF-GAA*, *trnfM-CAU*, *trnG-GCC**, *trnG-UCC*, *trnH-GUG*(×2), *trnI-CAU* (×2), *trnI-GAU**(×2), *trnK-UUU**, *trnL-CAA*(×2), *trnL-UAA**, *trnL-UAG*, *trnM-CAU*, *trnN-GUU*(×2), *trnP-UGG*, *trnQ-UUG*, *trnR-ACG*(×2), *trnR-UCU*, *trnS-GCU*, *trnS-GGA*, *trnS-UGA*, *trnT-GGU*, *trnT-UGU*, *trnV-GAC*(×2), *trnV-UAC**, *trnW-CCA*, *trnY-GUA*
Transfer RNAs	Subunit of acetyl-coA-carboxylase	*accD*
	c-type cytochrome synthesis gene	*ccsA*
	Envelop membrane protein	*cemA*
	Protease	*clpP***
	Translational initiation factor	*infA*
	Maturase	*matK*
Unknown function	Conserved open reading frames	*ycf1*(×2), *ycf2*(×2), *ycf3***, *ycf4*

Table S3. Variability of six hyper-variable regions in *Zingiber*.

Marker	Length (bp)	Variable base sites		Informative base sites		Nucleotide diversity (π)	Mean distance	Discrimination success (%)
		Number	Percentage (%)	Number	Percentage (%)			
rpl20	451	60	13.31	41	9.09	0.0199	0.0225	37.93
clpP	2217	91	4.11	47	2.11	0.0128	0.0073	65.52
ycf1	5914	481	8.13	281	4.75	0.0104	0.0146	58.62
rbcL-accD	1269	194	15.29	174	13.71	0.0277	0.0546	65.52
petA-psbJ	922	87	9.44	53	5.75	0.0389	0.0219	41.38
rpl32-trnL	990	111	11.21	63	6.36	0.0184	0.0195	48.28
Combined	11763	1024	8.71	659	5.61	0.0149	0.0175	86.21
